# The actions of exogenous leucine on mTOR signalling and amino acid transporters in human myotubes

**DOI:** 10.1186/1472-6793-11-10

**Published:** 2011-06-25

**Authors:** Petra Gran, David Cameron-Smith

**Affiliations:** 1 Molecular Nutrition Unit, School of Exercise and Nutrition Sciences, Deakin University, Burwood, Victoria, Australia

## Abstract

**Background:**

The branched-chain amino acid (BCAA) leucine has been identified to be a key regulator of skeletal muscle anabolism. Activation of anabolic signalling occurs via the mammalian target of rapamycin (mTOR) through an undefined mechanism. System A and L solute carriers transport essential amino acids across plasma membranes; however it remains unknown whether an exogenous supply of leucine regulates their gene expression. The aim of the present study was to investigate the effects of acute and chronic leucine stimulation of anabolic signalling and specific amino acid transporters, using cultured primary human skeletal muscle cells.

**Results:**

Human myotubes were treated with leucine, insulin or co-treated with leucine and insulin for 30 min, 3 h or 24 h. Activation of mTOR signalling kinases were examined, together with putative nutrient sensor human vacuolar protein sorting 34 (hVps34) and gene expression of selected amino acid transporters. Phosphorylation of mTOR and p70S6K was transiently increased following leucine exposure, independently to insulin. hVps34 protein expression was also significantly increased. However, genes encoding amino acid transporters were differentially regulated by insulin and not leucine.

**Conclusions:**

mTOR signalling is transiently activated by leucine within human myotubes independently of insulin stimulation. While this occurred in the absence of changes in gene expression of amino acid transporters, protein expression of hVps34 increased.

## Background

Amino acids are essential for the regulation of cell growth and proliferation [1,2] in two ways; by providing the substrate required for polypeptide biosynthesis, and by modulating signalling pathways responsible for protein synthesis [3-6]. Various cell models have examined the anabolic potential of the branched chain amino acid (BCAA), leucine, to stimulate skeletal muscle growth via mammalian target of rapamycin (mTOR) signalling [7,3,10]. Phosphorylation of mTOR complex 1, a rapamycin-sensitive kinase, is vital for downstream activation of phosphokinases required for translational initiation. Stimulation of mTOR by nutrients or insulin activates p70 ribosomal S6 kinase (p70S6K), a key mediator of the protein synthesis cascade [11]. Active p70S6K subsequently leads to phosphorylation of its downstream target, ribosomal protein S6 kinase (S6). This results in the translation of messenger RNA (mRNA) which encode for ribosomes and transcription factors, an essential process leading to increased cellular capacity to undergo protein synthesis [12]. Moreover, mTOR-catalysed stimulation of eukaryotic initiation factor 4E-binding protein (4E-BP1) results in its disassociation from eukaryotic initiation factor 4E (eIF4E). Subsequent binding of eIF4E to eIF4G forms the eIF4F translation initiation complex, and allows the recruitment of the 40S ribosomal subunit to the 5'-end of the mRNA to initiate protein translation [13].

Despite extensive evidence linking leucine with the activation of anabolic signalling [14,7-22,10], the proximal mechanisms by which mTOR responds to intracellular levels of leucine remain elusive. To date, several intermediary 'nutrient sensing' molecules, such as those characterised in *Saccharomyces cerevisiae *and bacteria, have been implicated in directing amino acid signalling to mTOR [23,24]. The ste-20 related mitogen-activated protein kinase kinase kinase kinase 3 (MAP4K3), activating proteins Rag 1-4 and class III phosphatidylinositol 3'-kinase vacuolar protein sorting 34 (Vps34) are thought to converge on signalling through mTOR in mammalian cells [25-27]. However, recent evidence from rodent models suggests that it is the activity of Vps34 which is the primary modulator of leucine-stimulated mTOR signalling in rat muscle [28]. Furthermore, it has been previously demonstrated that human vacuolar protein sorting 34 (hVps34) is required for nutrient activation of p70S6K, via mTOR signalling [29].

The intricate balance of amino acid influx and efflux in skeletal muscle is maintained by both system A and system L transport proteins, which are responsive to amino acid starvation and hormones [30-32]. Sodium coupled neutral amino acid transporter 2 (SNAT2), the predominant system A member expressed in human skeletal muscle, is reportedly regulated by both insulin and amino acid deprivation in cultured L6 myotubes [31,33]. Additionally, leucine exposure following serum withdrawal causes an enhanced rate of uptake of N-methylamino-alpha-isobutyric acid (MeAIB), a system A substrate, by SNAT2 [34]. However, it remains unknown whether enhanced uptake by SNAT2 occurs via increased gene transcription, augmented protein synthesis of the amino acid transporter or by increased activity. Similarly, system L-type amino acid transporters (LAT) have been extensively examined for their possible role in tumour growth, and exhibit a high affinity for BCAA [35,36]. Given the extensive evidence describing the potency of BCAA on mTOR-driven protein synthesis in skeletal muscle, no studies to date have examined the regulation of LAT transporters by amino acids, specifically BCAA. Identifying the effectiveness of leucine as a regulator of amino acid transporters within skeletal muscle will provide further insight in the role these proteins play in the control anabolic signalling.

Therefore in the present study, a human cell culture model was used to; (1) compare and contrast the effects of acute and chronic nutrient and hormonal stimulation of anabolic signalling which occurs in skeletal muscle, and (2) analyse their ability to modulate protein expression of the putative nutrient sensor, hVps34, and gene expression of selected amino acid transporters. It was hypothesised that leucine stimulated phosphorylation of mTOR-related signalling kinases would occur together with activation of hVps34 and amino acid transporter gene expression.

## Results

### Confirmation of a homogenous primary myotube culture

**Figure 1:**
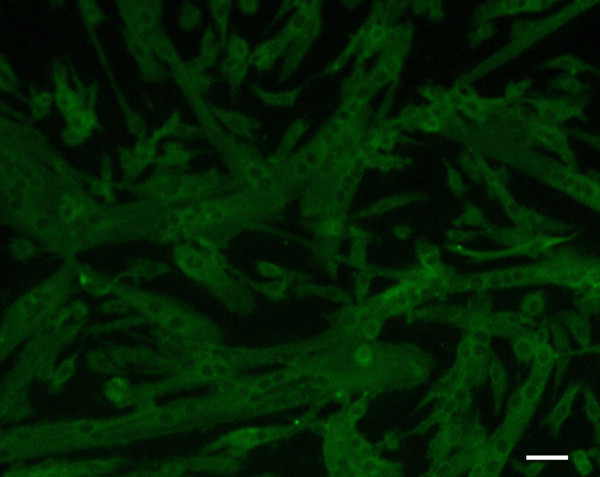
**Cultured human primary myotubes labelled for myogenin**. Primary myoblasts were grown to near confluence and induced to differentiate for 7 days. Myogenin (green) was detected by immunocytochemistry using anti-myogenin antibody followed by anti-mouse Alexa 488 secondary antibody. Scale bar = 30 μm.

Purity of primary myotube cultures was assessed by immunocytochemical labelling of the muscle cell marker myogenin (green; Figure 1). A typical purity of 95% was achieved.

### Leucine and insulin independently regulate mTOR signalling

**Figure 2:**
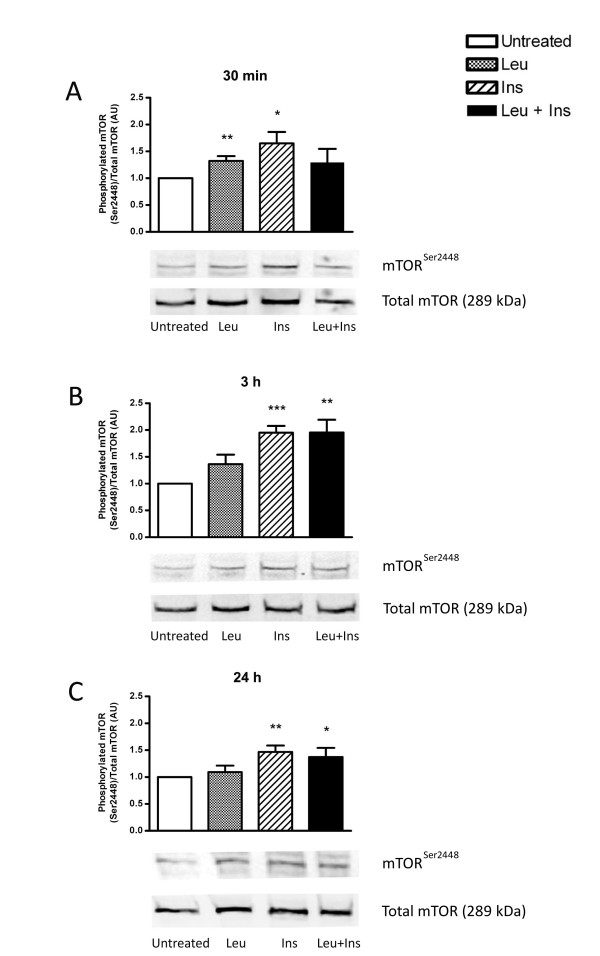
**Leucine and insulin independently regulate mTOR signalling**. Primary human myotubes were untreated or incubated in the presence of leucine (Leu; 5 mM), insulin (Ins; 100 nM) or co-treated with leucine and insulin (Leu + Ins) for 30 min (A), 3 h (B) or 24 h (C). Phosphorylation levels of mTOR at Ser2448 were determined by Western blot analysis relative to total levels of mTOR. Data are presented as mean ± SEM (n = 6). *P < 0.05, **P < 0.01, ***P < 0.001 versus untreated.

Leucine and insulin are known activators of mTOR in human skeletal muscle tissue. Thus the ability of these stimuli to regulate the expression of phosphorylated mTOR both individually and together in human myotubes was assessed (Figure 2). Cells treated with leucine or insulin for 30 min demonstrated a significant increase in phosphorylated serine mTOR following normalisation with total mTOR (1.3-fold, *P *< 0.01 and 1.6-fold; *P *< 0.05 respectively). Protein abundance of total mTOR did not change following treatment for all timepoints examined (Additional file 1, Figure S1). No evidence of synergistic activation of mTOR with combined leucine and insulin treatment was evident. Conversely, cultures stimulated for 3 h displayed increased mTOR expression under all treatment conditions, with the greatest increase observed with insulin stimulation (2.0-fold; *P *< 0.001). This was found to be maintained with co-treatment of leucine and insulin (2.0-fold; *P *< 0.01). At 24 h the response was diminished, with only insulin-treated myotubes exhibiting significant increases in mTOR phosphorylation (1.5-fold; *P *< 0.01), above baseline.

### Protein abundance of putative nutrient sensing protein, hVps34, is increased in response to leucine and insulin stimulation

**Figure 3:**
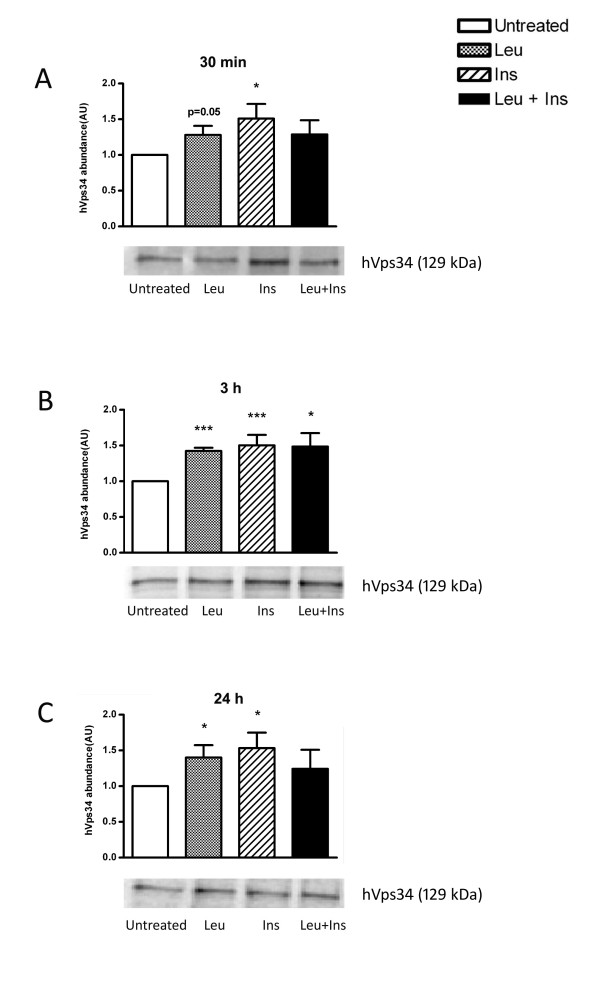
**Leucine and insulin supply modulate the protein abundance of hVps34**. Primary human myotubes were untreated or incubated in the presence of leucine (Leu; 5 mM), insulin (Ins; 100 nM or co-treated with leucine and insulin (Leu + Ins) for 30 min (A), 3 h (B) or 24 h (C). hVps34 protein abundance was determined by Western blot analysis. Data are presented as mean ± SEM (n = 6). *P < 0.05, ***P < 0.001 versus untreated.

It has been previously suggested that the activation of anabolic signalling is mediated through the nutrient-sensing protein, hVps34. In order to investigate whether the BCAA member, leucine, enhances hVps34 protein expression, hVps34 protein expression was measured in leucine and insulin-stimulated myotubes (Figure 3). Immunoblotting revealed that acute exposure (30 min) was sufficient to significantly increase hVps34 protein abundance in cells treated with leucine alone (1.3-fold; *P *= 0.05) and insulin alone (1.5-fold; *P *< 0.05). No synergistic effect occurred following co-treatment of leucine and insulin together. Similar increases were seen following 3 h stimulation of leucine (*P *< 0.001) and insulin (*P *< 0.001) with significant increases in hVps3 abundance occurring after co-stimulation of leucine with insulin (1.5-fold; *P *< 0.05). hVps34 protein expression remained elevated following incubation with leucine alone (*P *< 0.05) and insulin alone (*P *< 0.05) for 24 h.

### Leucine and insulin independently and synergistically regulate p70S6K signalling

**Figure 4:**
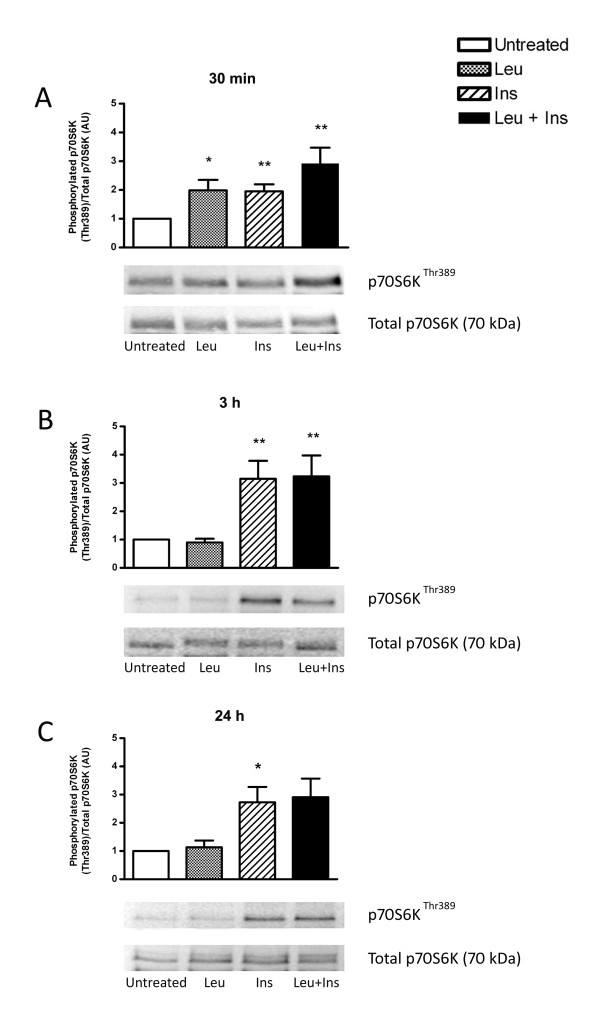
**Effect of leucine, insulin or in combination, on the phosphorylation state of p70S6K**. Primary human myotubes were untreated or incubated in the presence of leucine (Leu; 5 mM), insulin (Ins; 100 nM) or co-treated with leucine and insulin (Leu + Ins) for 30 min (A), 3 h (B) or 24 h (C). Phosphorylation levels of p70S6K at Thr389 were determined by Western blot analysis relative to total levels of p70S6K. Data are presented as mean ± SEM (n = 6). *P < 0.05, **P < 0.01, ***P < 0.001 versus untreated.

Activation of the downstream effector, p70S6K (Figure 4), occurred in response to both leucine (2.0-fold; *P *< 0.05) and insulin (2.0-fold; *P *< 0.01). Co-treatment with leucine and insulin resulted in synergistic activation of p70S6K (2.9-fold; *P *< 0.01) when normalised to total p70S6K. Interestingly, stimulation of leucine alone for 3 h did not affect levels of phosphorylated p70S6K on threonine 389, whereas insulin alone did (3.3-fold; *P *< 0.01). Similar observations occurred when myotubes were chronically treated for 24 h with insulin (2.7-fold; *P *< 0.05). No activation in response to leucine was detected following 24 h treatment.

### Leucine and insulin induce translation initiation via activation of eIF4G

**Figure 5:**
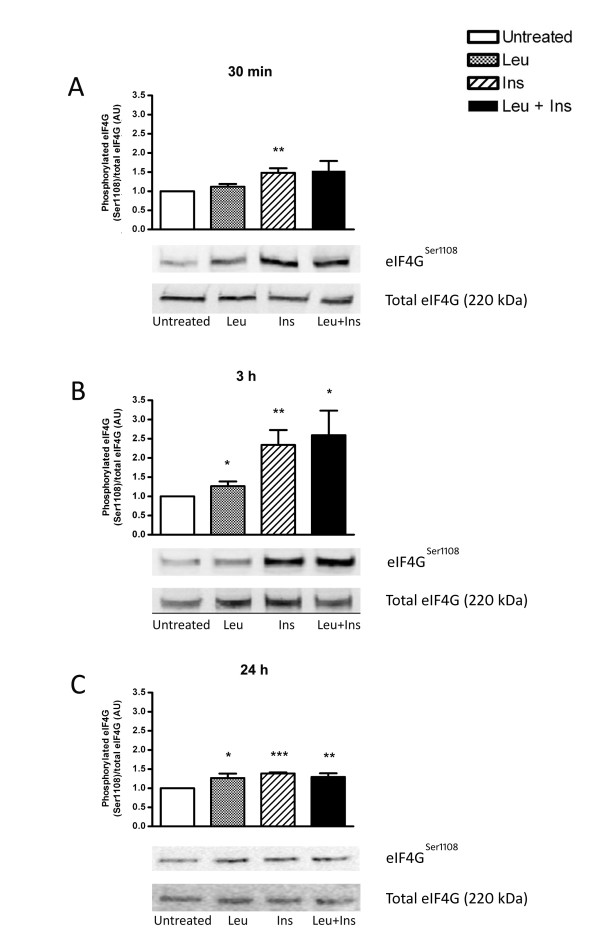
**Effect of leucine, insulin or in combination, on the activation of eIF4G**. Primary human myotubes were untreated or incubated in the presence of leucine (Leu; 5 mM), insulin (Ins; 100 nM) or co-treated with leucine and insulin (Leu + Ins) for 30 min (A), 3 h (B) or 24 h (C). Phosphorylation levels of eIF4G at Ser1108 were determined by Western blot analysis relative to total levels of eIF4G. Data are presented as mean ± SEM (n = 6). *P < 0.05, **P < 0.01, ***P < 0.001 versus untreated.

The effect of leucine and insulin on the activation of translation initiation was compared by measuring phosphorylation of eIF4G (Figure 5). Ser1108 phosphorylation significantly increased with 30 min insulin exposure (1.5-fold; *P *< 0.01) but remained unaltered upon exposure to leucine when normalised to total eIF4G. Contrastingly, exposure to leucine alone for both 3h and 24 h, and in combination with insulin, significantly stimulated signalling through eIF4G.

### Insulin but not leucine differentially regulates amino acid transporters

**Figure 6:**
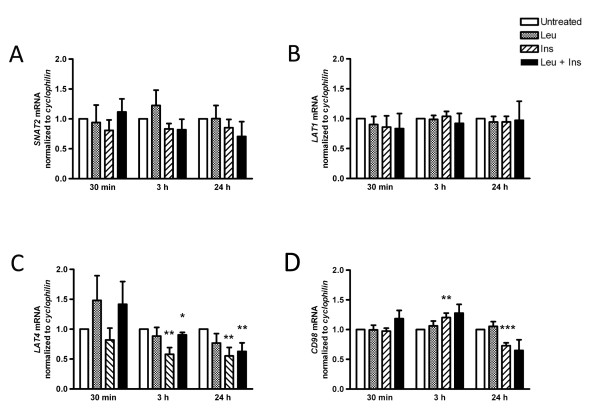
**Altered expressions of genes associated with amino acid transport**. The mRNA expression of *SNAT2 *(A), *LAT1 *(B), *LAT4 *(C) and associated glycoprotein *CD98hc *(D) was measured in primary human myotubes incubated with and without the presence of leucine (Leu; 5 mM), insulin (Ins; 100 nM) or co-treated with leucine and insulin (Leu + Ins) for periods indicated. Expression was detected by semi-quantitative real time polymerase chain reaction (qPCR) using specific oligonucleotide primers. Values are arbitrary units normalised to the expression levels of reference gene *cyclophilin *presented as means the means ± SEM (n = 6). *P < 0.05, **P < 0.01, ***P < 0.001 versus untreated.

To assess whether members of the system A and L transporter families undergo adaptive regulation following amino acid or insulin treatment, *SNAT2*, *LAT1*, *LAT4 *and *CD98hc *gene expression was measured (Figure 6). *SNAT2 *and *LAT1 *mRNA expression was not altered by leucine or insulin. Conversely, insulin alone or in combination with leucine resulted in a significant down-regulation of *LAT4 *at 3 h and 24 h. Insulin stimulation for 3 h significantly up-regulated *CD98hc *gene expression (1.2-fold, *P *< 0.01), however exposure for 24 h caused a significant decrease (0.3-fold, *P *< 0.001). *LAT4 *mRNA expression was unaltered by leucine treatment.

## Discussion

The aim of this study was to evaluate the anabolic effect of acute and chronic leucine and insulin stimulation in a model of human skeletal muscle. It was important to determine whether leucine could mediate mTOR-related signalling via activation of the putative regulator, hVps34, and amino acid transporters. The present work demonstrates the ability of the BCAA member, leucine, to independently stimulate mTOR signalling in an experimental model of human skeletal muscle. Although p70S6K was only transiently activated by leucine, chronic stimulation resulted in enhanced phosphorylation of the ternary kinase, eIF4G. Moreover, these data offer an explanation of the molecular mechanism by which amino acids modulate myogenic growth, via activation of the hVps34 protein. Finally, while exogenous leucine and insulin were sufficient to stimulate anabolic signalling in cultured myotubes, incubation did not alter gene expression of the amino acid transporters measured.

The use of human primary myotubes provides pertinent experimental data on amino acid-mediated anabolic signalling that occurs in human skeletal muscle tissue. Cultured muscle cells undergo differentiation to form mature myotubes [37] thus providing a physiologically relevant model to study molecular signalling events which occur *in vivo*. In agreement with data from rodent studies [17,14,9,20], Chinese hamster ovary cells (CHO) [38], pancreatic beta-cells [39] and rat hepatocytes [40], this study demonstrates for the first time that mTOR-related signalling kinases are responsive to a physiological dose of leucine in a human model of skeletal muscle, primary myotubes. However, while mTOR was stimulated by insulin on serine residue 2448, at all time points measured, the action of leucine was transient (1.3-fold increase) with phosphorylation diminishing after 30 min. This is consistent with Deldicque *et al. *(2008) who demonstrated a 1.5-fold increase in mTOR activation within C2C12 cells, a model of murine skeletal muscle, following an acute 30 min exposure to 5 mM leucine [17]. However, more recently it was reported that a 2 mM dose of leucine also stimulates mTOR phosphorylation on Ser2448 by a comparable magnitude [14]. The downstream biomarker, p70S6K, followed a similar trend to mTOR phosphorylation. While leucine demonstrated transient activation of p70S6K (2.0-fold increase), insulin appeared to stimulate this kinase at all time points. Previous work has demonstrated that 5 mM leucine stimulates p70S6K activation by 10-fold [17] and a 2 mM dose elicits a 5.9-fold increase [14] within rodent muscle cells. Furthermore, cultured pancreatic beta-cells have been shown to respond to 3.3 mM of leucine, therefore the stimulatory effect of leucine on the phosphorylation state of p70S6K may be specific to the cell model employed where by different cell types have a varying dose-response to amino acids [39]. Previous *in vitro *experiments demonstrates the synergistic activation of p70S6K in FAO cells treated with a combination of amino acids with insulin [41]. Results obtained from the current study did not exhibit an additive effect of leucine and insulin, despite utilising the same insulin concentration. While these experiments were conducted using leucine only, the former study used a mixture of amino acids. Furthermore, recent evidence suggests that while the activation of anabolic signalling is indeed mTOR-dependent, amino acids may signal through the Rag complex to activate mTORC1 while insulin requires the Ras homolog enriched in brain (Rheb) component to regulate anabolic signalling [42]. Moreover, it has also been previously shown that deletion of the Ser2448 phosphorylation site on mTOR did not affect mTOR activity and was not required for the downstream phosphorylation of PHAS-1 (4EBP1) or p70S6K in human embryonic kidney cells [43]. Therefore, it is possible that while mTOR phosphorylation in this study was transient, the downstream activation of p70S6K and eIF4G may occur through an independent signalling mechanism and requires further research.

Surprisingly, phosphorylation of eIF4G occurred following 3 h and 24 h treatment of leucine and insulin. Given that activation of eIF4G is a necessary step during formation of the translation initiation complex which is involved in cap dependent translation [13,44], activation by leucine indicates that protein synthesis may be initiated without insulin.

hVps34 is postulated to be a nutrient modulator responsible for signalling to mTOR. Recently, amino acid regulation of this class III PI3 kinase has been investigated in murine myotubes [28], HepG2 and CHO cells [29]. During states of amino acid starvation, hVps34 demonstrates reduced function. However, this is restored with the re-addition of amino acids [27,29]. Furthermore, previous reports demonstrate that endogenous expression of hVps34 is unresponsive to 1 μM insulin [29]. Unlike these studies, human primary muscle cells demonstrated increased hVps34 protein abundance following acute and chronic stimulation of leucine with and without 100 nM insulin. The response of hVps34 to insulin in cultured myotubes may reflect the physiological need of skeletal muscle for insulin-mediated glucose uptake and for muscle anabolism [45,46]. Limited studies have investigated the factors regulating hVps34 expression; however the possibility of cross-talk with insulin signalling requires further analysis.

Both system A and L solute carriers may regulate intracellular amino acid bioavailability within skeletal muscle [47]. The activity of system A transporters has been previously demonstrated to be insulin responsive cultured adipocytes, liver and rat muscle cells [48-50].

However, in cultured human myotubes both acute and chronic stimulation with insulin failed to increase *SNAT2 *and *LAT1 *gene expression. It is important to note that the aforementioned studies used supraphysiological doses of insulin, nearly 2-fold greater than the concentration used in the current study. System L transport proteins are essential for the movement of BCAA across the cell membrane of skeletal muscle cells [47]. Leucine exposure of human cultured primary muscle cells did not exert a change in either *LAT1 *or *LAT4 *gene expression, despite the specificity of LAT1 for leucine [51]. However, functional expression of LAT1 requires covalent association with the heavy chain of the CD98 antigen (CD98hc) in plasma membranes [52]. Acute insulin exposure stimulated an increase in CD98hc mRNA expression, however longer exposure resulted in suppressed gene expression. The current study aimed to present exploratory data on the regulation of amino acid transporters by leucine. Given the physiological need for amino acids in skeletal muscle, further research is required to delineate the factors affecting both the protein expression and cellular localisation of amino acid transporters.

## Conclusions

The present study provides the first evidence that mTOR signalling is enhanced in response to an acute stimulation with the proteinogenic amino acid, leucine, within cultured human myotubes. While these actions appear transient at the leucine dose utilised, activation of mTOR and p70S6K occurred at physiologically relevant concentrations independently of insulin stimulation. Interestingly, activation of mTOR signalling by leucine occurred in the absence of changes in the expression of genes encoding both the system A and system L carriers, which are responsible for amino acid transport. Thus, additional analyses are required to investigate the molecular mechanisms controlling amino acid transporter expression within skeletal muscle. Of note was the increased protein expression of hVps34, a putative leucine-sensitive kinase which intersects with mTOR. These results demonstrate the need for further clinical analysis to be performed specifically investigating the role of hVps34 as a nutrient sensing protein for mTOR signalling.

## Methods

### Subjects

Human primary skeletal muscle cells were obtained from the *vastus lateralis *muscle of six healthy male volunteers (24.6 ± 0.4 years). Informed written consent was obtained from each subject before participation in the study, after the nature, purpose and risks of the study were explained. All experimental procedures involved in this study were formally approved by the Deakin University Ethics Committee.

### Primary human skeletal muscle cell culture

Human primary skeletal muscle cells were cultured as previously described [53] using the percutaneous needle biopsy technique [37] modified to include suction [54]. The excised muscle was immersed and extensively washed in ice-cold Ham's F-10 medium (Gibco, Invitrogen Corporation, California, USA) containing 50 IU/ml penicillin, and 5 μg/ml streptomycin before being minced and digested in 0.5% Trypsin/EDTA (Gibco). The supernatant containing the myoblasts was then collected and the process repeated a further two times to break down any remaining tissue. Foetal bovine serum (FBS) (Gibco) was subsequently added to the supernatant to a final concentration of 10% (v/v). The supernatant was filtered through a 100 μm filter to remove any connective tissue and then spun to collect the cells. The resulting cell pellet was resuspended in primary growth medium (Hams F-10, 10% FBS, with 50 IU/mL penicillin, 5 μg/mL streptomycin and 2.5 pg/mL fibroblast growth factor-2 (FGF-2; Promega, Wisconsin, USA)) and then seeded on to an uncoated flask and incubated at 37°C for 30 minutes to induce fibroblast attachment, leaving myoblasts suspended in the medium. The medium was then transferred to second uncoated flask for a further 30 min to allow for any additional fibroblast attachment. The medium was aspirated and seeded on to an extracellular matrix-coated (ECM) (Sigma-Aldrich, New South Wales, Australia) flask. The resulting primary cell cultures were maintained in primary growth medium in humidified air at 37°C containing 5% CO_2_. To determine culture homogeneity, the expression of specific myogenic markers (myoD, myogenin and desmin) was analysed using immunocytochemistry (Figure 1). In all experiments, the minimal myogenic purity was 80% for myoblast preparations [55].

### Leucine and insulin stimulation experiments

When cells reached 70% confluence, proliferation medium was replaced with differentiation medium containing 2% horse serum. Following 7 d of differentiation, cells were serum starved overnight (16 h) prior to treatment. Myotubes were then exposed to serum-free media containing an additional 5 mM of water-soluble L-leucine (Calbiochem, California, USA) ± human insulin (100 nM; Actrapid^®^; Novo Nordisk, Australia) for either 30 min, 3 h or 24 h. Five millimolar was selected as an appropriate concentration for these experiments as dose-response studies demonstrated that 5 mM L-leucine achieved the greatest stimulatory effect (data not shown). All experiments were conducted on cells at passage five.

### Protein extraction and immunoblot analysis of insulin signalling kinases

Cells were lysed after 30 min, 3 h and 24 h, in lysis buffer (pH 7.0) containing 20 mM Tris-HCl, 5 mM EDTA, 10 mM Na-pyrophosphate, 100 mM NaF, 2 mM Na_3_VO_4_, 1% Igepal, 10 μg/mL Aprotinin, 10 μg/mL Leupeptin, 3 mM Benzamidine and 1 mM PMSF. The supernatants were rotated for 1 h at 4°C and subsequently centrifuged at 14,000 *rpm *for 15 min with the resulting supernatant collected into a fresh tube. Protein concentrations were determined using a BCA Protein Assay (Pierce, Thermo Scientific, New South Wales, Australia) with bovine serum albumin (BSA) as standard.

Protein lysates were denatured in loading buffer containing dithiothreitol (DTT) and separated by sodium dodecyl sulphate-polyacrylamide gel electrophoresis (SDS-PAGE). Proteins were then transferred onto nitrocellulose membrane and blocked at room temperature using 5% (w/v) BSA (Sigma-Aldrich) in tris buffered saline with 0.1% (v/v) Tween-20 (TBST; Sigma-Aldrich). The following phospho-specific antibodies were added and incubated overnight at 4°C in blocking buffer: mTOR^Ser2448^, p70S6K^Thr389^, eIF4G^Ser1108 ^(Cell Signaling, Massachusetts, USA) and hVps34 (Santa Cruz). Membranes were subsequently washed and then incubated with anti-rabbit HRP-conjugated secondary antibody (1:1000; Calbiochem) for 1 h at room temperature. Following this, membranes were washed repeatedly as before, and proteins visualised using enhanced chemiluminescence (Perkin-Elmer, Queensland, Australia) on a Kodak 4000MM Image Station (Kodak, New York, USA) using a CCD camera. Band density was quantified using Kodak imaging software version 4.5.0 (Kodak). For normalisation, blots were stripped using Restore Western Blot Stripping buffer™ (Quantum Scientific, Victoria, Australia) and then subsequently reprobed for total mTOR, total p70S6K and total eIF4G proteins to verify the relative amount of analysed proteins.

### RNA extraction

RNA was extracted from primary myotubes using TRI-reagent (Ambion Inc., Texas, USA) after the treatment period. Chloroform was added to separate phases. Following centrifugation for 15 min at 14,000 *rpm*, the aqueous layer was removed and an equal volume of isopropanol added and incubated at -20°C for 1 h. Following RNA precipitation, samples were centrifuged at 14,000 *rpm *to pellet the RNA. The pellet was washed with 75% ethanol in diethyl pyrocarbonate (DEPC)-treated water and then resuspended in nuclease free water (NFW). Total RNA concentration and quality were determined using the Nanodrop ND-1000 (Nanodrop Technologies, Delaware, USA).

### cDNA synthesis

First strand cDNA was generated from 1 μg total RNA using the High Capacity RNA-to-cDNA kit (Applied Biosystems) according to manufacturer's instructions. Briefly, a 20 μL reaction mixture consisting of 1 μg of total RNA, 10 μL of 2X RT Buffer Reaction Mix, 1 μL of 20X RT Enzyme Mix and a known amount of NFW and placed into the thermocycler (Hybaid, Thermo Scientific) with the following temperature and time protocol: 37°C for 60 min, 95°C for 5 min, and 4°C for 5 min. All synthesised cDNA samples were stored at -20°C until further analysis.

### Semi-quantitative real time PCR (qPCR)

Analysis of gene expression was performed using the Applied Biosystems 7500 Real-Time PCR system (Applied Biosystems). cDNA (diluted 1:20) was analysed using SYBR green fluorescence (Power SYBR Green Master Mix, Applied Biosystems) and with primer pairs specific for the gene of interest. Oligonucleotide primers were designed using Primer Express 3.0 (Applied Biosystems) software and validated using BLAST sequence alignment and in the absence of primer-dimers and secondary structures. All oligonucleotides were purchased from Geneworks (South Australia, Australia). Refer to Table 1 for primer sequences.

**Table 1:** Details of primer sequences used in qPCR analysis

Gene of interest	Genbank Accession Numbers	Sequences of primers	Amplicon length (bp)
*Cyclophilin*	NM_021130.3	FP: 5' CATCTGCACTGCCAAGACTGA 3'	72
		RP: 5' TTCATGCCTTCTTTCACTTTGC 3'	
*SNAT2*	NM_018976.4	FP: 5' TGCTGTGCCAATTCTGATCTTT 3'	185
		RP: 5' TGAGACTATGACGCCACCAACT 3'	
*LAT1*	NM_003486.5	FP: 5' AGGAGCCTTCCTTTCTCCTG 3'	181
		RP: 5' CTGCAAACCCTAAGGCAGAG 3'	
*LAT4*	NM_152346.1	FP: 5' TCTCTCCGTGCTCATCTTCA 3'	165
		RP: 5' ATTCCTGGAAAGGTGACTGC 3'	
*CD98hc*	NM_002394.5	FP: 5' GCAGATCGACCCCAATTTTG 3'	79

Each reaction contained 8 μL of 2X Sybr green master mix, 2 μL of forward primer, 2 μL of reverse primer, 6 μL sterile nuclease-free water and 2 μL of cDNA (1.25 ng/μL). All samples were run in triplicate. An initial cycle of 10 min at 95°C was used to denature the cDNA, followed by 40 cycles consisting of 95°C for 15 s and subsequent extension at 60°C for 1 min.

### Reference gene normalisation and calculations

Data were analysed using comparative critical threshold (Ct) method where the amount of target normalised to the amount of endogenous control is given by 2^-ΔΔCt ^(Applied Biosystems). The efficacy of *cyclophilin *(CYC) as an endogenous control was examined using the equation 2^-ΔCt^. It was considered an appropriate endogenous control for this study because no changes in the expression of this gene were observed (data not shown).

### Statistical analysis

Statistical analysis was performed using SPSS version 17.0 for Windows (SPSS Inc., Illinois, USA). Data are reported as means ± standard error of the mean (SEM). All data sets were tested for normal distribution and variance homogeneity using Levene's test for Equality of Variances. A Student's t-test was used to determine significance between the treatment groups. A probability level of <0.05 was adopted throughout to determine statistical significance unless otherwise stated.

## Abbreviations

BCAA: branched-chain amino acid; hVps34: human vacuolar protein sorting 34; LAT: L-type amino acid transporter; mTOR: mammalian target of rapamycin; mRNA: messenger RNA; p70S6K: p70 ribosomal S6 kinase; SNAT2: sodium coupled neutral amino acid transporter 2

## Competing interests

The authors declare that they have no competing interests.

## Authors' contributions

PG conducted all experiments, statistical analyses and drafted the manuscript. DCS conceived the study, participated in its design and coordination and helped to draft the manuscript. Both authors read and approved the final manuscript.

## Supplementary Material

Additional file 1 **Protein abundance of total mTOR is not affected by either leucine or insulin**. A figure representing the protein expression of total mTOR at 30 min, 3 h and 24 h. Primary human myotubes were untreated or incubated in the presence of leucine (Leu; 5 mM), insulin (Ins; 100 nM) or co-treated with leucine and insulin (Leu + Ins) for 30 min (A), 3 h (B) or 24 h (C). Protein abundance of total mTOR was determined by Western blot analysis. Data are presented as mean ± SEM (n = 6). Click here for file
