# Supplementary figures and images for: The actions of exogenous leucine on mTOR signalling and amino acid transporters in human myotubes
Source: BMC Physiol. 2011 Jun 25;11:10. doi: 10.1186/1472-6793-11-10 (PMC3141572; doi:10.1186/1472-6793-11-10)

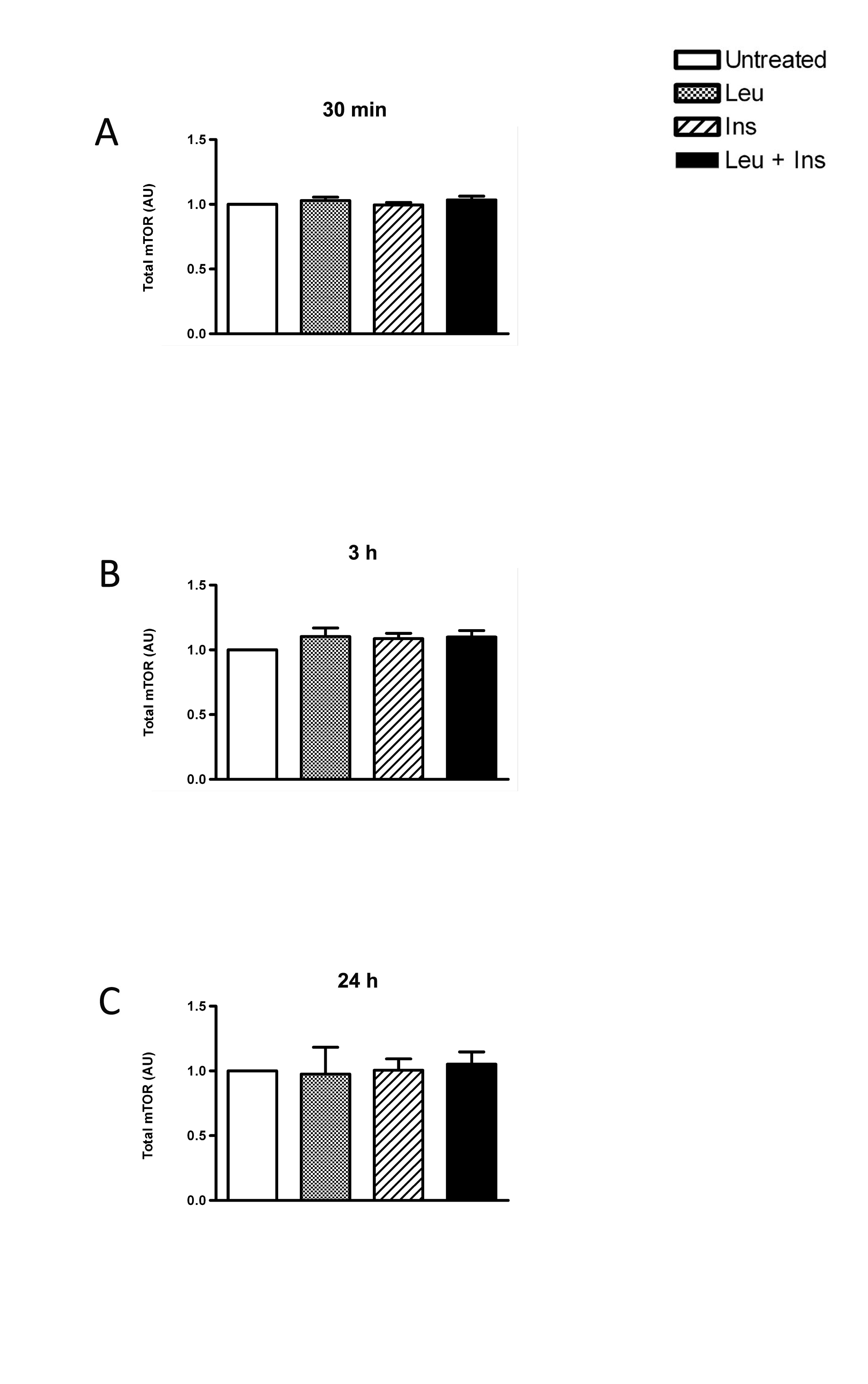

Supplement: Additional file 1 — Protein abundance of total mTOR is not affected by either leucine or insulin. A figure representing the protein expression of total mTOR at 30 min, 3 h and 24 h. Primary human myotubes were untreated or incubated in the presence of leucine (Leu; 5 mM), insulin (Ins; 100 nM) or co-treated with leucine and insulin (Leu + Ins) for 30 min (A), 3 h (B) or 24 h (C). Protein abundance of total mTOR was determined by Western blot analysis. Data are presented as mean ± SEM (n = 6). [file 1472-6793-11-10-S1.JPEG]
